# Sudden Increases in Listeriosis Rates in England and Wales, 2001 and 2003

**DOI:** 10.3201/eid1503.071432

**Published:** 2009-03

**Authors:** Benjamin J. Cairns, Robert J.H. Payne

**Affiliations:** University of Bristol, Bristol, UK

**Keywords:** Listeria, foot and mouth disease, seasonality, temperature, England, Wales, dispatch

## Abstract

The monthly incidence of listeriosis infections in England and Wales had 2 sudden increases during April 2001 (41%) and March 2003 (48%). Although no causative association is demonstrated, these increases correspond to key dates relating to the onset and aftermath of the 2001 foot and mouth disease outbreak in the United Kingdom.

Prevention of listeriosis (infection caused by *Listeria monocytogenes*) is a serious food safety issue, particularly for pregnant women, the elderly, and those who are immunocompromised. Death occurs in 20%–30% of cases, making listeriosis a leading cause of food poisoning deaths in Europe and the United States (*1*). An increasing rate of listeriosis has been reported in several European countries (*2*). Our study focused on the large increase in the number of reported listeriosis cases in England and Wales during 2 months in separate years (April 2001 and March 2003). These increases were permanent and cumulative; after each increase, monthly incidence of listeriosis did not return to previous levels. These increases primarily reflect a higher rate of bacteremic listeriosis in those >60 years of age and are not otherwise correlated with geography, gender, ethnicity, socioeconomic factors, or infectious serotypes (*3*).

## The Study

We compared monthly listeriosis data from England and Wales with temperature records from 1989 through 2007 to determine the influence of various potential predictors on the number of listeriosis cases. UK Health Protection Agency (HPA) data listing total monthly cases of human listeriosis in England and Wales during 1990–2007 (*4**,**5*) are aggregate. All age categories and regions were included and were collated by the HPA Centre for Infections from voluntary reporting by microbiology laboratories and from referrals of cultures. These publicly available data were also validated by the HPA, and in our analysis we used revised figures based on that validation. Our analysis covered the period from 1990, when active surveillance of listeriosis began, through 2007. Pregnancy-associated cases (mother and neonate) were counted as 1 case. Undated cases that could not be assigned to a particular month were excluded from analysis. We used the UK Met Office mean monthly area temperature time series for 1989–2007 and 30-year means averaged during 1961–1990 (*6*).

**Figure 1:**
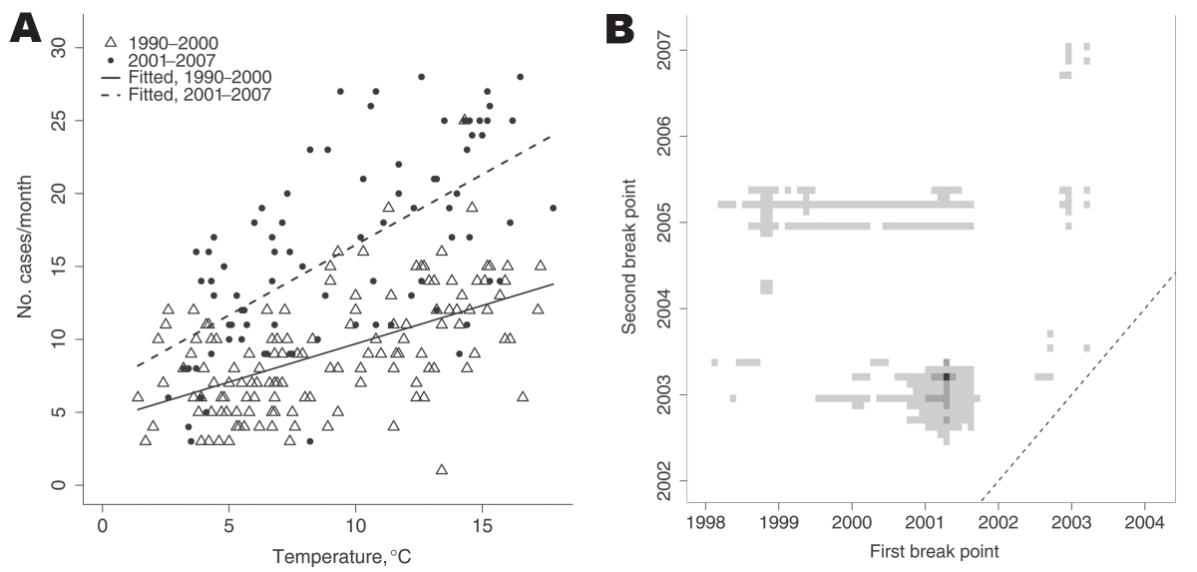
Exploratory analyses of changing rates of listeriosis. A) Listeriosis cases compared with mean observed monthly UK temperatures, 1990–2000 (triangles) and 2001–2007 (circles). Shown are an increased overall incidence in 2001–2007 (dashed line) versus 1990–2000 (solid line) and a significant change in the linear dependence of incidence on temperature (p = 0.001). B) Best-fit pair of break points and other pairs of break points with support, according to the corrected Akaike Information Criterion (AIC). The 2 break-point months are varied to find the lowest value (black square). Pairs of break points with good support relative to the best model (corrected AIC within 4 of the best fit; dark gray squares) or moderate to weak support (corrected AIC within 10 of the best fit; light gray squares) are also shown. Pairs of break points with little or no support (corrected AIC >10 greater than the best fit; white area) include those models for which only 1 break point exists (squares along the dashed line).

Exploratory linear regression analyses suggested a positive correlation between the number of listeriosis cases and the monthly mean UK ambient temperature, as well as suggesting a change in this relationship after 2000 (p = 0.001; Figure 1, panel A). However, residual variability was not constant, and the monthly counts are likely to be overdispersed due to clustering of cases (*3*). The data were fitted again by using a negative binomial generalized linear model with a logarithmic link function, a common model for time series of foodborne illness cases (*7**,**8*). To separate seasonality of listeriosis rates from dependence on temperature, we considered the 30-year mean monthly temperatures from 1961–1990, as well as monthly temperature anomalies (observed mean temperature minus 30-year mean). To determine whether temperatures could have a delayed effect on listeriosis incidence, we also included mean and anomaly temperature variables lagged by 1 or more months. To allow for 2 break points at which the incidence of listeriosis may have suddenly changed, dummy variables were used to represent periods before, between, and after the months in which these increases might have occurred. A best-fit model was selected according to the corrected Akaike Information Criterion (AIC) (*9*) by using stepwise regression at all combinations of 2 break point months from January 1996 through December 2007. To examine changing effects on incidence, interactions between break point indicators and other variables were considered, even if the main effects were not yet in the model. Main effects were subsequently added to the final model if any interaction terms were included.

**Figure 2:**
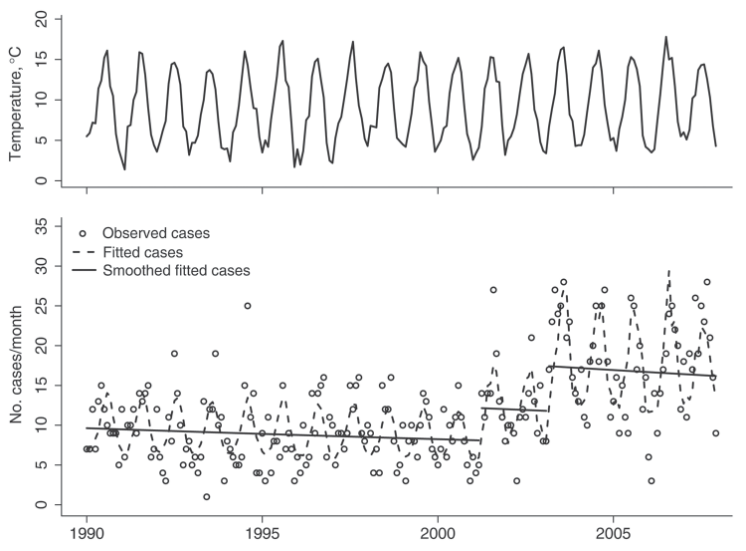
Monthly listeriosis cases and temperature observations, England and Wales, 1990–2007. The monthly number of listeriosis cases (circles, lower plot) is strongly seasonal, with a close relationship to the monthly mean temperature (solid line, upper plot). Overall listeriosis incidence per month underwent 2 sudden increases, at break points around April 2001 and March 2003. Our fitted statistical model (dashed line, lower plot) provides a close fit to the observed data; the seasonally-adjusted fitted model (solid line, lower plot) shows the large magnitudes of the jumps in the rate of cases at the 2 break points.

The best-fit model included 2 break points (Tables 1, 2; Figure 1, panel B; Figure 2). The overall rate of cases increased by 40.83% in April 2001 (adjusted p = 0.001, 95% confidence interval [CI] 17.29%–68.93%); a further increase of 47.76% occurred in March 2003 (adjusted p<0.001, 95% CI 27.92%–71.13%). According to the corrected AIC, there is statistical evidence of changes in the incidence of listeriosis in a range of months around these best-fit values, and weak support for a change in early 2005, apparently due to low numbers of cases in the first months of that year and the following winter (Figure 1, panel B). Most of the seasonality in the number of cases was accounted for by the 30-year mean monthly temperatures. Each extra degree Celsius of mean monthly temperature corresponded to a 2.42% increase in cases in the current month (adjusted p = 0.044, 95% CI, 0.55%–4.33%) and a 4.09% increase in the following month (adjusted p<0.001, 95% CI 2.19%–6.03%). The 1-month lag reflects delays between food production and consumption plus the known long incubation of listeria infections. Similar lags have been observed for other enteric pathogens (*6**,**11*). A relationship also appears to exist between the rate of listeriosis and the 1-month lagged temperature anomaly starting in April 2001, which corresponded to an additional 5.71% of listeriosis cases per degree Celsius (95% CI –2.10%–14.15%) when compared with the main effect of temperature anomaly in 1990–2007. This increase was not significant (adjusted p = 0.312), although the power of this test is only ≈15% at an adjusted significance level of 0.05 (post hoc power analysis performed by computer simulation of the fitted model in the R statistical computing environment). The model also includes a slight negative trend in cases over time (–0.13% per month, 95% CI –0.26%–0.01%), but evidence for this trend is very weak (adjusted p = 0.188).

**Table 1:** Coefficient names and descriptions for the best-fit negative binomial generalized linear model of listeriosis incidence

Variable	Coefficient description
(Intercept)	Log of overall monthly no. cases in March 2001
GEAPR01	Change in log cases (April 2001)
GEMAR03	Change in log cases (March 2003)
MEANTEMP	Log cases/°C 30-year mean temperature of the current month
MEANTEMP1	Log cases/°C 30-year mean temperature of the previous month
MONTHNUM	Overall linear trend of log cases/month
ANOMALY1	Overall log cases/°C previous month’s temperature anomaly
ANOMALY1×GEAPR01	Change in log cases/°C previous month's temperature anomaly from April 2001
θ (theta)	Negative binomial response distribution size parameter

**Table 2:** Estimated coefficients for terms in the best fit model for the monthly incidence of listeriosis*†

Variable	Coefficient	95% Confidence interval	Unadjusted p value	Adjusted p value
(Intercept)	1.5430	1.3961–1.6879	<0.001	<0.001
GEAPR01	0.3424	0.1594–0.5243	<0.001	0.001
GEMAR03	0.3904	0.2462– 0.5373	<0.001	<0.001
MEANTEMP	0.0239	0.0055–0.0424	0.011	0.044
MEANTEMP1	0.0401	0.0217–0.0586	<0.001	<0.001
MONTHNUM	–0.0013	–0.0027–0.0001	0.063	0.188
ANOMALY1	0.0246	–0.0246–0.0739	0.327	0.327
ANOMALY1×GEAPR01	0.0556	–0.0212–0.1323	0.156	0.312
θ (theta)	600.41	NA	NA	NA

*NA, not applicable. †p values are for 2-sided tests of significance of the difference of each coefficient from zero; adjusted p values are according to Holm’s method (*10*). Percentage changes in the main text can be derived from these coefficients by taking exponentials, subtracting 1, and multiplying by 100.

The changes in the incidence of listeriosis appear to have been quite sudden (Figure 2). Causative explanations based on gradual demographic or behavioral changes have previously been ruled out (*3*), and those based on dynamic processes seem unlikely because no evidence exists for epidemiologic feedback between the source of infections and clinical cases. One possibility is that the increases are due to contamination of a small number of food products, as has been suggested as an explanation for an upsurge in listeriosis rates in the late 1980s. However, a restricted range of strains was responsible for most of the additional cases at that time (*12*), and no evidence exists of such a pattern since 2001 (*3*). An alternative possibility is that the phenomenon is a consequence of changes in government policy or business practices that have had more widespread effects on food processing, distribution, or preparation.

We found notable coincidences between the dates of the increases in listeriosis infection rates and the dates of events associated with the 2001 foot and mouth disease (FMD) outbreak in the United Kingdom. The April 2001 increase in listeriosis rates occurred shortly after the outbreak of FMD in February 2001, allowing for a delay similar to the incubation period of listeria infections. The March 2003 increase in listeriosis rates occurred in the same month as the relaxation of movement restrictions on livestock instituted after the 2001 FMD outbreak (*13*). The diverse and widespread consequences of the 2001 FMD outbreak are well-documented (*14**,**15*), and it seems plausible that such major disturbances to agricultural production could be the ultimate cause of the large increases in listeriosis rates that have been observed. The coincidence of these events raises the possibility that the change in listeriosis rates would be an unrecognized outcome of the 2001 UK FMD crisis, although we caution that our analysis of the of these cases does not demonstrate whether a causative link exists.

## Conclusions

Listeriosis incidence in England and Wales has increased notably since the beginning of 2001, with 2 separate, sudden increases recorded in April 2001 and March 2003. Gillespie et al. argue that blame for these increases cannot be ascribed to any of a variety of specific factors (*3*). Instead, more widespread changes affecting food production, processing, or consumption could be the root of the problem. Incidence of this serious disease has risen substantially in England and Wales, and an understanding of why will be important for management of listeriosis as a public health issue.
